# Cinnamaldehyde-modified chitosan hybrid nanoparticles for DOX delivering to produce synergistic anti-tumor effects

**DOI:** 10.3389/fbioe.2022.968065

**Published:** 2022-10-11

**Authors:** Zuoqin Zhou, Caiyun Wang, Jingqi Bai, Zihan Zeng, Xiaoyu Yang, Bing Wei, Zheng Yang

**Affiliations:** ^1^ Research Center of Anti-aging Chinese Herbal Medicine of Anhui Province, School of Biology and Food Engineering, Fuyang Normal University, Fuyang, China; ^2^ Anhui Ecological Fermentation Engineering Research Center for Functional Fruit Beverage, School of Biology and Food Engineering, Fuyang Normal University, Fuyang, China; ^3^ Engineering Research Center of Biomass Conversion and Pollution Prevention of Anhui Educational Institutions, School of Chemistry and Materials Engineering, Fuyang Normal University, Fuyang, China

**Keywords:** chitosan, lactobionic acid, cinnamaldehyde, anti-tumor, drug carrier

## Abstract

Cancer cells are under oxidative stress associated with the increased generation of reactive oxygen species (ROS). Therefore, increasing the oxidative stress of tumor cells by delivering ROS generators is an effective strategy to induce apoptosis of cancer cells. Herein, we reported a hybrid nanoparticle based on lactobionic acid (LA) modified chitosan and cinnamaldehyde (CA) modified chitosan, which possesses both active tumor-targeting ability and ROS regulation ability, in order to have a synergistic effect with the anti-tumor drug doxorubicin (DOX). LA can improve the tumor-targeting ability and cellular accumulation of these nanoparticles, and CA can induce apoptotic cell death through ROS generation, mitochondrial permeability transition and caspase activation. The particle size and distribution as well as drug release profiles of these nanoparticles were observed. *In vitro* and *in vivo* antitumor studies demonstrated that the hybrid nanoparticles show a significant synergistic antitumor effect. Thus, we anticipate that the hybrid nanoparticles have promising potential as an anticancer drug carrier.

## 1 Introduction

Cancer is still one of the most serious diseases that threaten human life (Calado et al., 2018). In recent years, with the development of targeted therapy and immunotherapy, the clinical treatment of cancer patients has improved significantly (Cui et al., 2018). However, during chemotherapy, cancer cells evolve and acquire “multidrug resistance” (MDR), which severely limits the efficacy of cancer treatment and affects the survival and quality of life of patients (Cheng et al., 2015; Gurunathan et al., 2018). MDR is defined as the development of resistance to one drug, which ultimately leads to resistance to many different drugs, which are different in structure and function from the original drug. Many studies have demonstrated that multidrug resistance occurs within a short period time after taking certain anticancer drugs (Wang Z et al., 2020). The mechanism of MDR may refer to the over-expressed drug efflux transporters (such as P-glycoprotein and MDR-associated proteins); the upregulated glutathione/glutathione S-transferase (GSH/GST) detoxification system; the repairing of damaged DNA and anti-apoptosis mediated by Bcl-2 activation (Cheng et al., 2015; Gurunathan et al., 2018; Hou et al., 2019; He et al., 2020). Lots of corresponding therapeutic strategies have been developed to overcome MDR.

Recently, the strategy of enhancing chemotherapy and overcoming MDR by adjusting the content of reactive oxygen species (ROS) in cancer cells has received attention from researchers, which is named as “oxidation therapy.” ROS is one of a majority intracellular metabolites, including hydrogen peroxide (H_2_O_2_), superoxide (O^2−^), hydroxyl radicals (−OH), which play important roles in the growth, proliferation and signal transduction of normal cells (Chuang et al., 2012; Kim et al., 2013; Cui et al., 2018; Dong et al., 2018; Pan et al., 2019). Compare with normal cells, the abnormal cancer cells are under oxidative stress, which is related to the increase in ROS production caused by the destruction of ROS homeostasis. Although cancer cells can suffer from this oxidative stress to a certain extent, when the level of reactive oxygen species exceeds the tolerance threshold of these cells, it will lead to apoptosis and necrosis of cancer cells (Noh et al., 2015; Li et al., 2017a; Wei et al., 2017; Dong et al., 2018; He et al., 2020; Li et al., 2020; Li and Kataoka, 2021). Therefore, the oxidative stress state makes cancer cells more sensitive to exogenous ROS generators and antioxidant inhibitors (Dong et al., 2018). The typical method of “oxidation therapy” is to deliver ROS-generating agents to cancer cells directly, including glucose oxidase (GOx), catalase (CAT), methyl quinone (MQ) and cinnamaldehyde (CA) (Noh et al., 2015; Zhen et al., 2018; Fu et al., 2019; Xu et al., 2021). Another approach of oxidation therapy is to disrupt the redox balance in cancer cells by suppressing the intracellular concentration of GSH (Noh et al., 2015; Li et al., 2016; Li et al., 2017b; Cui et al., 2018; Jiang et al., 2021; Xu et al., 2021).

Cinnamaldehyde is a bioactive compound isolated from cinnamon, which is approved by Food and Drug Administration (FDA) for using as a food additive (Kim et al., 2013; Cheng et al., 2015; Mateen et al., 2019; Qu et al., 2019). Various studies indicated that CA has anti-bacterial activities, anti-fungal activities and immunomodulating functions. Many researches have also demonstrated that CA can inhibit cancer cells and induce apoptotic cell death through ROS generation, reduce mitochondrial membrane potential, release cytochrome C, and caspases activation. Despite its potential antitumor ability, the low solubility and weak stability (half-life of ∼4 min) of CA and lack of specificity to tumor tissues severely limit its clinical translation (Kim et al., 2013; Gurunathan et al., 2018; Wang Z et al., 2020; Wang X et al., 2020; Xu et al., 2021). To address this issue, nano-drug delivery systems (NDDS) have been developed for physical loading or covalent binding CA, avoiding the direct contact with blood and prolonging the half-life of CA. Lee reported a CA-based polymeric prodrug, which further self-assembled with camptothecin (CPT) to form a dual acid-responsive micelles. *In vitro* and *in vivo* studies demonstrated that the micelles can induce apoptotic cell death by the generation of ROS and display significant synergistic antitumor effect with CPT (Kim et al., 2013). Tang developed a carrier-free nano-drug based on 5-fluorouracil (5FU) and cinnamaldehyde (CA) conjugated prodrug. The self-assembled 5FU-CA nanoparticles had higher tumor growth inhibition than 5FU and CA mixture and exhibited lower systemic toxicity under the same reducing dose of each drug [Self-assembled 5-fluorouracil-cinnamaldehyde nanodrugs for greatly improved chemotherapy *in vivo*] (Fang et al., 2021). Therefore, the combination of CA and chemotherapeutics can significantly increase the level of ROS in cancer cells, amplify oxidative stress, and further produce a significant synergistic effect with chemotherapeutics.

**SCHEME 1:**
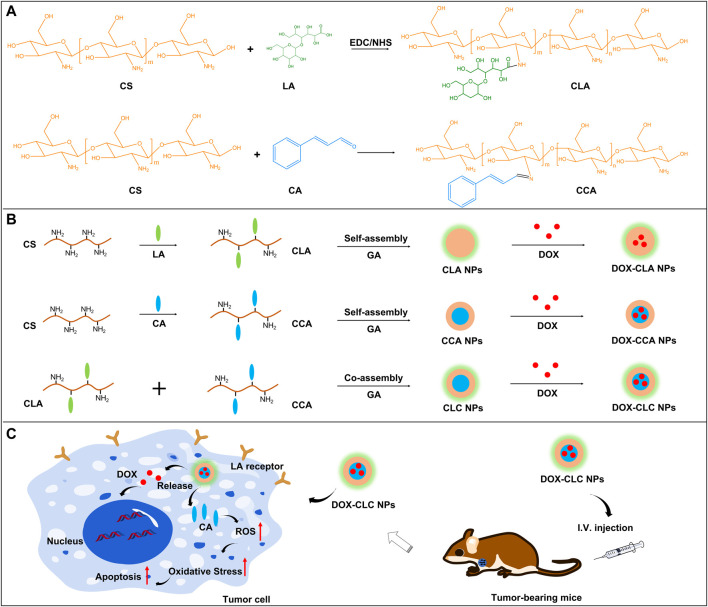
The synthesis routes and chemical structure of CLA and CCA **(A)**; The preparation of DOX-CLA NPs, DOX-CCA NPs and DOX-CLC NPs **(B)**; LA-based active targeting and synergistic antitumor process of CA and DOX **(C)**.

In this study, we reported a hybrid nanoparticle with both active tumor-targeting ability and ROS regulation ability, in order to have a synergistic effect with the anti-tumor drug (Scheme 1). Firstly, CA-modified chitosan (CCA) was prepared by the reaction of the aldehyde group of CA and the amino group of chitosan. CCA was then mixed with lactobionic acid (LA) modified chitosan (CLA) to prepare the hybrid nanoparticles (CLC NPs). CLC NPs are further loaded with the anti-tumor drug doxorubicin (DOX), to give drug-loaded nanoparticles (CLC-DOX NPs). It has been proved that the LA-modified chitosan nanoparticles have good tumor targeting ability and can effectively improve the accumulation of DOX in the tumor tissues (Wei et al., 2017). After cellular uptake of CLC-DOX NPs, the Schiff base breaks under acidic conditions, releasing CA and DOX. CA can induce apoptosis of cancer cells by the generation of ROS and produce a synergistic anti-tumor effect with DOX. Thus, the rationally designed hybrid CLC-DOX NPs have dual modes of anticancer actions with synergistic therapeutic effects.

## 2 Materials and methods

### 2.1 Materials

Chitosan (CS, MW:3,000 Da; Deacetylation degree >90%) was purchased from Bomei Biotech Co., Ltd. (Hefei, China). N-(3-dimethylaminopropy)-N′-ethylcarbodiimide hydrochloride (EDC), N-hydroxysuccinimide (NHS), Cinnamaldehyde (CA), Fluorescein isothiocyanate (FITC) and lactobionic acid (LA) were obtained from Mackin Biochemical Co., Ltd. (Shanghai, China). Doxorubicin hydrochloride (DOX) was purchased from Meilun Biotechnology Company (Dalian, China). 3-(4,5-dimethyl-2-thiazolyl)-2,5-diphenyl-2-H-tetrazolium bromide (MTT) was obtained from Sigma Chemical Co.,. Ltd. Human liver carcinoma (HepG2) and reactive oxygen detection kit (DCFH-DA) were purchased from KeyGEN BioTECH (Nanjing, China).

### 2.2 Methods

#### 2.2.1 Synthesis of LA–conjugated chitosan

LA modified chitosan was prepared as our previous work (Wei et al., 2017). In brief, 10 mg of LA was dissolved in 10 ml deionized water and activated with EDC (64.2 mg) and NHS (39 mg) under stirring for 1 h. Next, a certain amount of LA solution was added into chitosan solution (20 mg/ml) and reacted at room temperature for 24 h. The mixture solution was then dialyzed against deionized water for 48 h to remove unreacted LA and EDC/NHS.

#### 2.2.2 Synthesis of CA–conjugated chitosan

CA modified chitosan was prepared as previous work. Briefly, 100 mg of CS was dissolved in 10 ml of deionized water, CA (30 μl) was then added to the above solution, and reacted for 12 h under stirring. After the reaction, the solution was transferred to a dialysis bag (MWCO 3,500 Da) and dialyzed against deionized water for 24 h to remove unreacted CA. The product was lyophilized to obtain pure CCA powder.

#### 2.2.3 Preparation of CLA and CCA based nanoparticles

Three kinds of nanoparticles were prepared by a desolvation method. The nanoparticles based on CLA were named CLA NPs, the nanoparticles based on CCA were named CCA NPs, and the hybrid nanoparticles prepared based on CLA and CCA were named CLC NPs. CLA or CCA was dissolved in deionized water at the concentrations of 10 mg/ml at room temperature. To this solution, a certain amount of ethanol (ethanol/water = 2:1) was added to give a cloudy mixture, indicating the formation of CLA or CCA nanoparticles. The nanoparticles were then cross–linked by glutaraldehyde solution (20 μl) for 6 h. The mixture solution was dialyzed against deionized water for 24 h to give pure CLA or CCA NPs. Dissolved CLA and CCA together in deionized water, the molar ratio of CLA to CCA was set as 1:4. Next, add an appropriate amount of ethanol to promote the formation of nanoparticles. After cross-linking with GA for 6 h, a pure nanoparticles solution was obtained by dialysis for 24 h, named as CLC NPs. The size distributions and polydispersity index (PDI) of these nanoparticles were measured by dynamic light scattering (Zeta Sizer Nano Series, Malvern, United Kingdom). FITC-labeled nanoparticles were prepared as follows: 0.5 mg of FITC was dissolved in 1.0 ml DMSO, and then added into 3 ml of CLA NPs or CCA NPs or CLC NPs. After reaction for 12 h, the nanoparticles were transferred into a dialysis bag (MWCO 14 kDa) and dialyzed against deionized water for 14 h to remove unreacted FITC. The FITC-labeled nanoparticles were named as FITC-CLA NPs, FITC-CCA NPs and FITC-CLC NPs.

#### 2.2.4 Preparation of DOX–loaded NPs

DOX was then loaded into these nanoparticles prepared in the previous step to obtain corresponding drug-loaded nanoparticles. The process is as follows: A certain amount of DOX solution (10 mg/ml in deionized water) was added into 2 ml of CLA NPs or CCA NPs or CLC NPs solution, the mass ratio of DOX to nanoparticles was set as 1:3, 1:5, and 1:10, respectively. The mixed solution was stirred and reacted for 8 h in the dark at room temperature. After the reaction, the mixture was centrifuged at 0.8 × 10^4^ rpm for 10 min to remove the unloaded DOX, and the precipitate was re-dispersed in 2 ml of deionized water to obtain DOX-loaded nanoparticles. The corresponding nanoparticles were designed as DOX-CLA NPs, DOX-CCA NPs and DOX-CLC NPs. The size of these DOX-loaded nanoparticles was measured by DLS. The micromorphology of DOX-CLA NPs, DOX-CCA NPs and DOX-CLC NPs were observed by scanning electron microscope (SEM). Each sample was diluted to proper concentration and placed onto a silicon wafer and coated with a thin layer of gold (20 s) before observed by SEM (S-4800, Japan). The concentration of un–loaded DOX in the supernatant was determined by UV spectrophotometer at 481 nm. The drug loading content (DLC) and drug loading efficiency (DLE) were calculated using following formulas:

DLC(%)=(weight of DOX in NPs)/(weight of NPs)×100%

DLE(%)=(weight of DOX in NPs)/(weight of freeing drug)×100%

#### 2.2.5 *In vitro* DOX release at different pH values

The drug release ability of these DOX-loaded nanoparticles was measured at pH 5.0, 6.0, and 7.4. The process was as follows: 2 ml of DOX-CLA NPs or DOX-CCA NPs or DOX-CLC NPs suspension was added into a dialysis bag (MWCO 3500 Da), the concentration of DOX was set as 0.5 mg/ml. The dialysis bags were then immersed in 10 ml of 0.01 M PBS solution, and shake it at 100 rpm in the dark. At the predesigned time (1, 2, 4, 8, 12, 24, 48, 72, 96, and 120 h), 10 ml of the release medium was withdrawn, and replaced with 10 ml of fresh PBS. DOX concentration in the release medium was measured by a microplate system (spectraMax M2e Molecular devices, United States) at an excitation wavelength of 480 nm and an emission wavelength of 590 nm. The cumulative amount of DOX released from each sample was calculated and plotted against time.

#### 2.2.6 The determination of cellular uptake

H22 cells (Mouse liver cancer cell line) and HepG2 cells (Human hepatocarcinoma cell line) were cultured in RPMI 1640 that contained 10% FBS, 1% penicillin (100 U/ml) and streptomycin (0.1 mg/ml). H22 cells and HepG2 cells were seeded in a confocal laser dish for 24 h at 37°C in a humidified atmosphere of 5% CO_2_. The initial cell concentration is 5 × 10^5^ cells/dish. Then, cells were co-cultured with 0.2 ml of free DOX, DOX-CLA NPs, DOX-CCA NPs, and DOX-CLC NPs, respectively. The DOX concentration in the culture medium was set as 8 μg/ml. After incubating for 4 h, HepG2 cells were then washed with PBS three times, fixed with 4% paraformaldehyde (PFA) for 10 min, and stained with Hoechst 33258 for 10 min. H22 is a non-adherent cell. The cultured H22 cells were collected by centrifugation (1,000 rpm, 5 min), re-dispersed in fresh PBS, and repeated three times to remove free DOX or nanoparticles in the culture medium. H22 cells were then fixed with 4% PFA for 10 min and stained with Hoechst 33258 for 10 min. Finally, HepG2 or H22 cells were observed by confocal laser scanning microscopy (CLSM; FV1000, Olympus). Besides, free LA (0.5 mg/ml) was incubated with HepG2 cells to saturate the LA receptor on the cell membrane surface, and then co-cultured the FITC-labeled nanoparticles with these cells for 4 h. Cells not incubated with LA were directly co-cultured with nanoparticles for 4 h as a control. The intracellular response intensity was further observed by CLSM. The FITC fluorescent intensity in these cells were measured by ImageJ.

#### 2.2.7 *In vitro* ROS evaluation of each sample in HepG2 cells

ROS generation was assessed using a reactive oxygen detection kit (DCFH-DA). Briefly, DCFH-DA was diluted with cell culture medium in a ratio of 1:1,000. HepG2 cells (5 × 10^5^ cells/well) were co-incubated with free DOX, DOX-CLA NPs, DOX-CCA NPs, and DOX-CLC NPs at a DOX concentration of 8 μg/ml. After 4 h, cell culture medium was replaced and DCFH-DA (1 ml) solution was added and co-incubated for 30 min. The cells were washed with PBS solution three times to remove the free DCFH-DA. Next, cells were fixed with 1 ml PFA before CLSM observation. The fluorescent intensity in these cells were measured by ImageJ.

#### 2.2.8 *In vitro* cytotoxicity of these DOX-loaded NPs

*In vitro* cytotoxicity of empty nanoparticles and DOX-loaded nanoparticles were evaluated by MTT assay with H22 and HepG2 cell lines. In brief, H22 or HepG2 cells were seeded in 96-well plates at a density of 5 × 10^3^/well and incubated for 24 h. Next, cells were co-cultured with CLA NPs, CCA NPs, CLC NPs, free DOX, DOX-CLA NPS, DOX-CCA NPs, and DOX-CLC NPs at various concentrations for 48 h, respectively. The DOX concentration in each sample was set as 1, 2, 4, 8, and 16 μg/ml. After incubating for 48 h, the culture medium with each sample was removed, and replaced with180 μl fresh culture medium and 20 μl MTT (5 mg/ml in PBS solution) for another incubation of 4 h. Finally, the medium was removed again, 150 μl DMSO was added to dissolve formazan crystals from living cells. The absorbance of each well was measured at 570 nm by a microplate reader (spectraMax M2e Molecular devices).

#### 2.2.9 Growth inhibition in tumor like multicellular spheroids

HepG2 based multicellular spheroids (MCS) were cultured as previously reported work [13]. Briefly, 5 ml cells suspension containing 5 × 10^5^ HepG2 cells was added into a poly-HEMA-coated cell culture flask and cultured in humidified atmosphere with 5% CO2. HepG2 MCS with the size around of 150 μm were spontaneously formed within 3 days. Next, several spheroids with a diameter around 150 μm were incubated with free DOX, DOX-CLA NPs, DOX-CCA NPs and DOX-CLC NP, respectively. The DOX concentration was set as 16 μg/ml. The culture medium with each sample was changed every other day. The change of morphology and size of MCS during the experiment were observed by an inverted microscope at day 1, day 3, day 5, and day 7. The average diameter changes of MCs during the treatment were measured by ImageJ.

#### 2.2.10 *In vivo* antitumor activity

To investigate the antitumor effect of DOX-CLC NPs, H22 tumor-bearing mice were established and *in vivo* antitumor studies were processed as follows: 1 × 10^6^ H22 cells suspended in 0.2 ml of saline were inoculated into the left armpit of ICR male mice (22–25 g). Mice were then divided into three groups (6 mice/group) randomly when the tumor volume reached 100–150 mm^3^. 0.2 ml of saline (control group), free DOX (6 mg/kg), DOX-CLC NPs (6 mg/kg, eq.) were administrated I.V., and this day was set as day 1. The body weight and tumor size of these mice were measured every other day, and the volume of tumor was calculated as the following equation:

$$V=d^{2}\times D/2$$

Where “*D*” is the maximum of tumor and “*d*” is the minimum of tumor. The antitumor experiment was observed for 14 days. At the end of this study, each mouse was executed and tumor mass was collected, weighed and imaged.

Tumor tissues were then fixed with paraformaldehyde solution (4%) for 2 days and implanted in paraffin. Each section was cut into 5 μm slices, and processed for hematoxylin and eosin (H&E) staining. To further study the antitumor mechanism of nanoparticles, the apoptotic state of tumor cells was characterized by TUNEL staining. Tumor sections were investigated under an inverted fluorescence microscope. To observe the toxic effects of the prepared NPs, major organs such as the heart, liver, spleen, lungs, and kidneys were also stained with H&E according to the standard procedure. All these in vivo experiments were performed in accordance with the guidelines established by the Animal Care Committee of Anhui Medical University.

#### 2.2.11 Statistical analysis

Student’s *t*-test was employed to determine the difference in MCs inhibition and tumor inhibition. * represents *p* < 0.05, ** represents *p* < 0.01; *p* values less than 0.05 were considered statistically significant.

## 3 Results and discussion

### 3.1 Preparation and characterization of CLA NPs, CCA NPs, and CLC NPs

**FIGURE 1:**
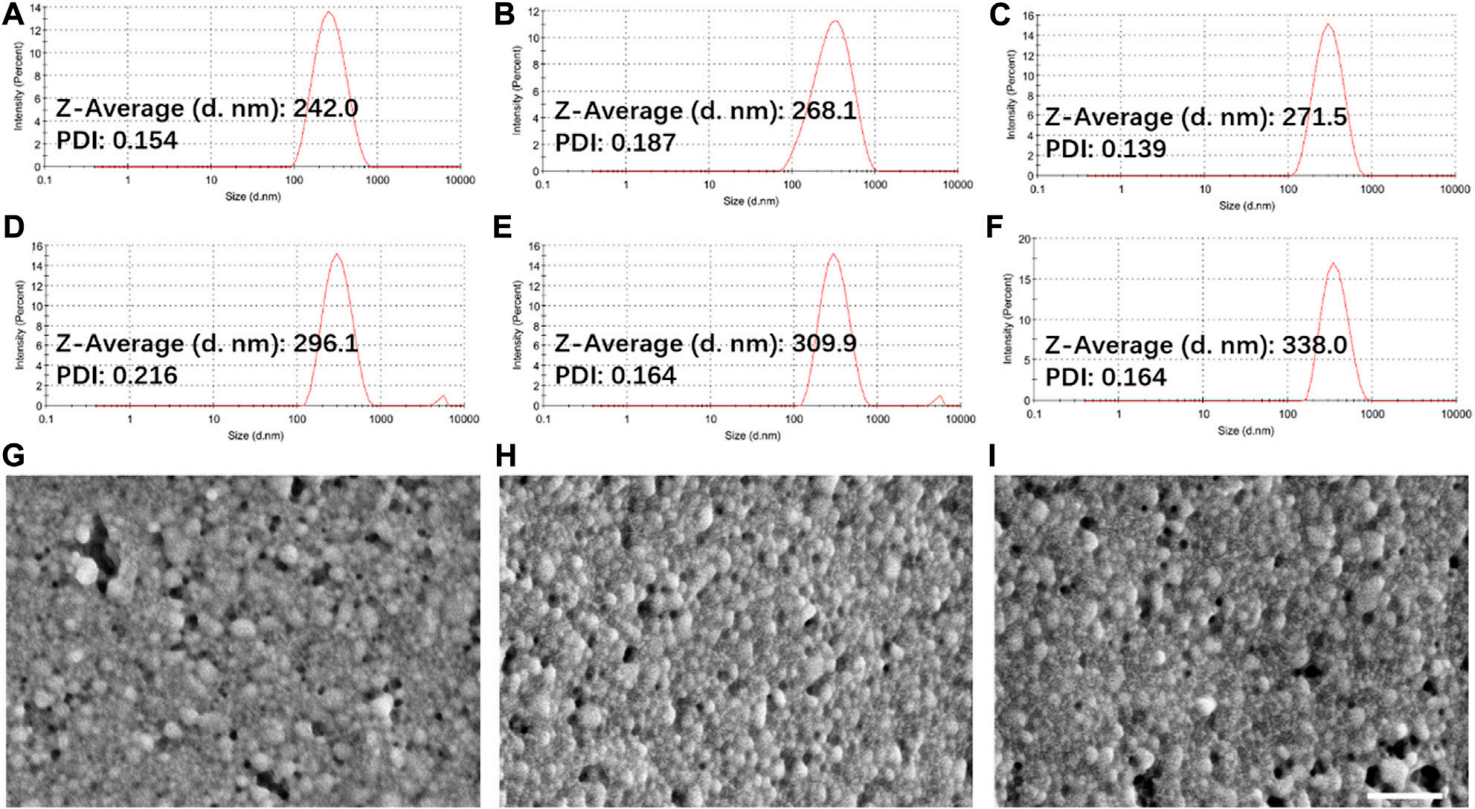
Particle size and PDI of CLA NPs **(A)**, CCA NPs **(B)**, CLC NPs **(C)**, DOX-CLA NPs **(D)**, DOX-CCA NPs **(E)** and DOX-CLC NPs **(F)** measured by DLS; SEM images of DOX-CLA NPs **(G)**, DOX-CCA NPs **(H)**, and DOX-CLC NPs **(I)**; Scale bar = 500 nm.

Herein, a hybrid nanoparticle with both tumor targeting and cancer cell suppression functions was prepared based on lactobionic acid (LA) modified chitosan and cinnamaldehyde (CA) modified chitosan. The synthesis route and chemical structure as well as the antitumor mechanism of the hybrid nanoparticles were shown in Scheme 1. As shown in Scheme 1: LA modified chitosan (CLA) was obtained through EDC/NHS reaction, and CA modified chitosan (CCA) was obtained through Schiff base reaction. Next, CLA or CCA can self-assemble to nanoparticles in an aqueous solution, to give CLA NPs and CCA NPs, respectively. CLA and CCA were blended and self-assembled in aqueous solution at a molar ratio of 1:4 to obtain hybrid nanoparticles (CLC NPs). CLA NPs, CCA NPs and CLC NPs were further crosslinked by GA to improve their stability. Finally, all these nanoparticles are further loaded with the chemotherapeutic drug doxorubicin (DOX) to obtain corresponding drug-loaded nanoparticles, DOX-CLA NPs, DOX-CCA NPs and DOX-CLC NPs. LA, as a tumor-targeting ligand, can enhance the cellular uptake and drug delivery efficiency of these nanoparticles, while CA can kill tumor cells directly by the generation of ROS, thereby producing a synergistic anti-tumor effect with DOX. The particle size and polydispersity index (PDI) of these nanoparticles were then observed by DLS, and the results were presented in Figure 1. The hydrolysis diameter of CLA NPs, CCA NPs and CLC NPs measured by DLS were 242.0, 268.1, and 271.5 nm, while the PDI were 0.154, 0.187, and 0.139, respectively. After loading with DOX, the particle size of the three kinds of nanoparticles increased to a certain extent. The particle size of DOX-CLA NPs, DOX-CCA NPs and DOX-CLC NPs were 296.1, 309.9, and 338.0 nm, while the corresponding PDI were 0.216, 0.164, and 0.164, respectively. The increase in particle size may be due to the adsorption of DOX on the surface of the nanoparticles. In addition, the micromorphology of DOX-CLA NPs, DOX-CCA NPs and DOX-CLC NPs were observed by SEM. It can be found that the three nanoparticles are spherical, and the particle size is smaller than the result of DLS detection, which may be caused by the dehydration of nanoparticles during the preparation of SEM samples. However, the drug-loaded nanoparticles still have an appropriate particle size and good dispersibility, which is conducive to drug delivery in the tumor area.

### 3.2 *In vitro* DOX release at different pH values

**FIGURE 2:**
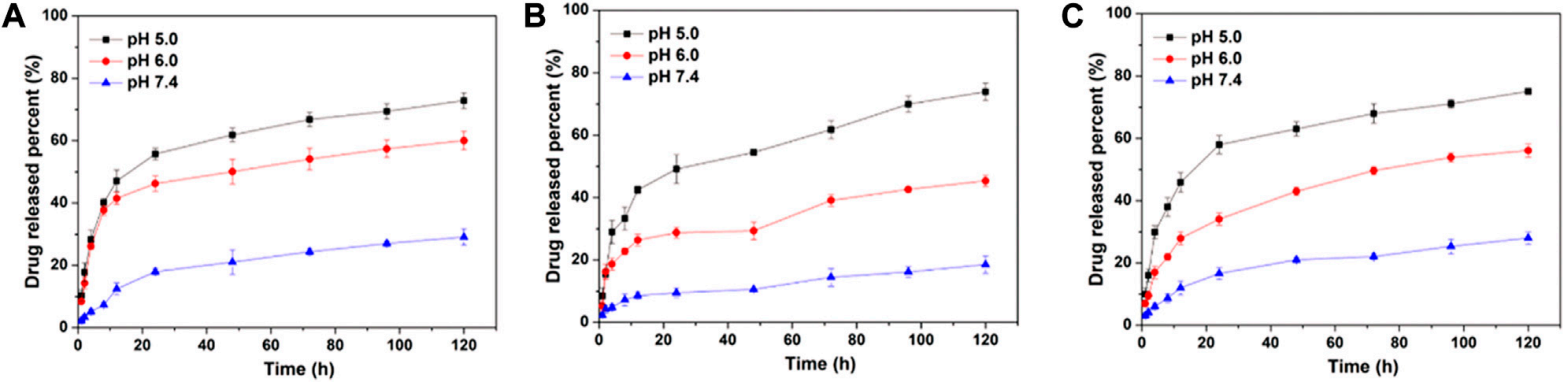
*In vitro* DOX release from DOX-CLA NPs **(A)**, DOX-CCA NPs **(B)**, and DOX-CLC NPs **(C)** at pH 7.4, pH 6.0, and pH 5.0.

NDDS is usually injected into the blood circulation through intravenous injection and then enriched in tumor tissues through the EPR effect. Therefore, nanoparticles should be able to maintain stability in the blood without releasing drugs, but quickly release drugs in tumor tissues or cancer cells. Considering the pH gradient between normal tissues and tumor tissues, DOX release profiles of DOX-CLA NPs, DOX-CCA NPs and DOX-CLC NPs were detected at pH 7.4, 6.0 and 5.0, and the results were displayed in Figure 2. Firstly, all these DOX-loaded nanoparticles show similar drug release trends, which may be due to that the main component of the carrier material is composed of chitosan and the three particles have similar particle sizes. In addition, the DOX release rate of DOX-CLA NPs, DOX-CCA NPs, and DOX-CLC NPs were all very slow at pH 7.4. For DOX-CLA NPs, only 12.5% of DOX was released after 12 h, and 29.1% of DOX was released after 120 h. For DOX-CCA NPs, the 8.6% of DOX was released within 12 h and 18.5% of DOX was released within 120 h. The corresponding DOX release amount of DOX-CLC NPs was 12.0% and 28.0%, respectively. This result indicates that the prepared nanoparticles can remain stable under pH 7.4, prevent the unmatured drug release, and can improve drug delivery efficiency while reducing its toxicity to normal tissues. However, there is still a small amount of DOX released from the nanoparticles, which may be caused by the re-dissolution of the DOX adsorbed on the outer-layer of the nanoparticles. In addition, all these NDDS exhibits a pH-dependent drug release behavior, and the amount of DOX released increases rapidly with the decrease of pH values. During the experiment, 60.0% of DOX released from DOX-CLA NPs under pH 6.0, while up to 72.9% of DOX released from DOX-CLA NPs under pH 5.0. At pH 6.0 the DOX release amount of DOX-CCA NPs is 45.4%, while the release amount of DOX-CLC NPs is 56.1%. Within 120 h, the DOX release amount from DOX-CCA NPs at pH 5.0 is 73.9%, while the corresponding result of DOX-CLC NPs is 75.1%. The reasons for the faster drug release rate as the pH decreases may include: the solubility of DOX increases as the pH decreases; the amino groups of chitosan and doxorubicin are protonated at low pH, and the electrostatic repulsion between DOX and carrier is destroyed. Thus, the results demonstrated that the prepared nanoparticles do not release drugs at pH 7.4, but quickly release loaded drugs under acidic conditions, which is beneficial to improve the efficacy of anti-tumor drugs and reduce their serious side effects.

### 3.3 Cellular uptake and ROS detection

**FIGURE 3:**
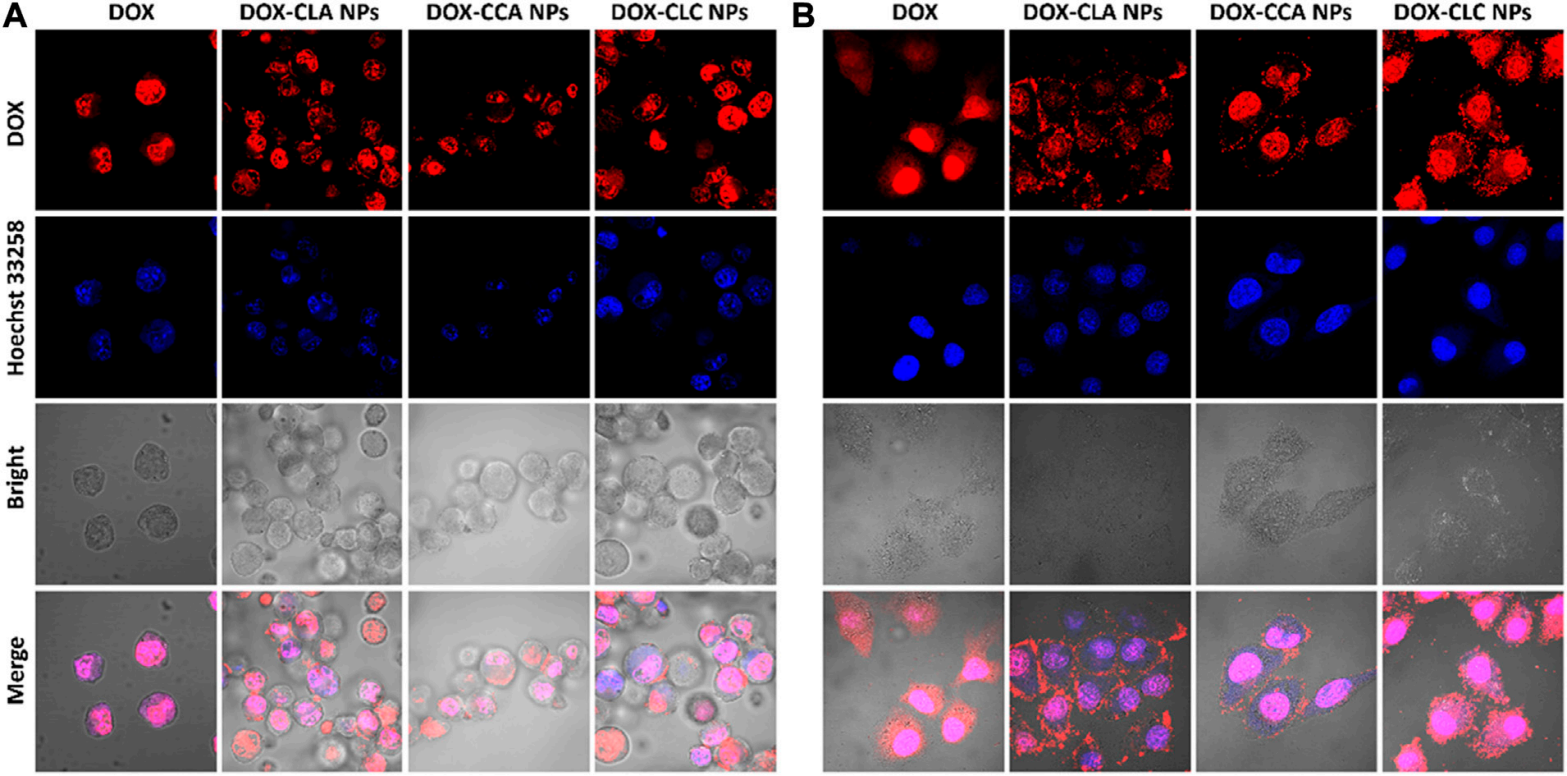
Cellular uptake of free DOX, DOX-CLA NPs, DOX-CCA NPs and DOX-CLC NPs after co-cultured with H22 cells **(A)** and HepG2 cells **(B)** for 4 h.

**FIGURE 4:**
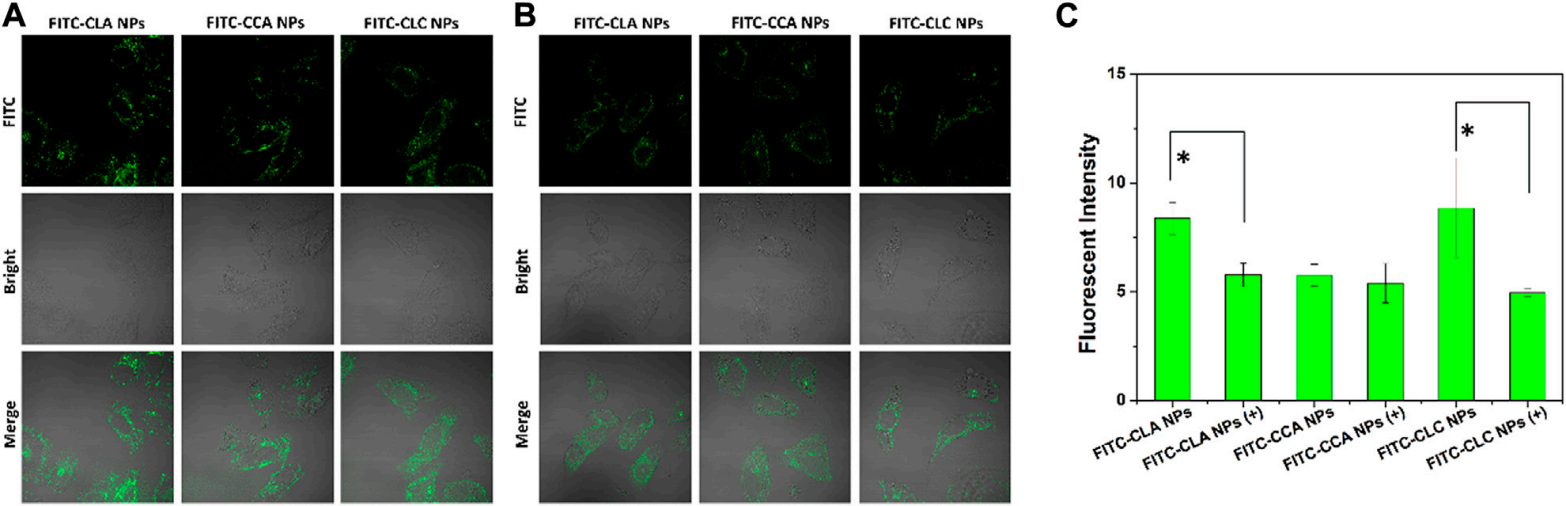
FITC-labeled nanoparticles co-incubated with HepG2 cells for 4 h **(A)**; Incubate cells with free LA for 30 min to saturate the LA-receptor, and then co-incubate cells with FITC-labeled nanoparticles for 4 h **(B)**; Quantitative analysis of the FITC fluorescence intensity measured by ImageJ **(C)**, +means cells were pre-incubated with LA; * represents *p* < 0.05; ** represents *p* < 0.01.

Cellular uptake and rapid release of drugs in cells are very important for NDDS to realize their antitumor effect. Thus, free DOX and DOX-loaded nanoparticles were co-cultured with H22 cells and HepG2 cells for 4 h, and the cellular uptake and DOX distribution were measured by CLSM. Figure 3 shows the cellular uptake of H22 and HepG2 cells co-cultured with free DOX, DOX-CLA NPs, DOX-CCA NPs and DOX-CLC NPs for 4 h. For H22 cells, it was found that a weak red signal occupied in cell nucleus after incubated with free DOX, indicating that free DOX can cross the cell membrane and enter cell nucleus by passive diffusion. The results of cellular uptake and distribution of DOX-loaded nanoparticles are different from those of free DOX: The obvious granular red signal in the cytoplasm means that the nanoparticles enter the cell by endocytosis; meanwhile, the diffuse red signal appears in the cytoplasm, indicating that the nanoparticles release the drug in the cell, and the free DOX further diffuses into the nucleus. The cellular uptake by HepG2 cells is similar with those of H22 cells. All these results show that although the mechanisms of free DOX and DOX-loaded nanoparticles entering cells are different, nanoparticles can indeed effectively deliver DOX into cells to produce cytotoxicity. To further verify the targeting function of LA, we co-cultured free LA and HepG2 cells to saturate the LA receptor on the cell membrane, and then co-cultured FITC-labeled particles and cells for 4 h, and used CLSM to observe the presence/absence of free LA. Effect of LA preincubation on cellular uptake of nanoparticles. It can be seen from Figure 4A that there are a lot of green fluorescent signals in the cytoplasm, but no corresponding signals in the nucleus, indicating that the LA-modified chitosan nanoparticles can enter the cells well. As can be seen from Figure 4B, the fluorescence signals in the cytoplasm of each group of particles decreased significantly, indicating that pre-incubated cells with free LA saturated the LA receptors, thereby blocking the uptake of nanoparticles to a certain extent. In addition, the targeting function of LA should be further quantitatively analyzed by flow cytometry. The fluorescent intensity in Figures 4A,B were measured by ImageJ, and the results were shown in Figure 4C. From the results, it can be seen that the intracellular fluorescence intensity of nanoparticles modified with LA (DOX-CLA NPs and DOX-CLC NPs) is higher than that of nanoparticles without LA (DOX-CCA NPs). On the other hand, when cells were pre-incubated with free LA, the intracellular fluorescence intensity of DOX-CLA NPs and DOX-CLC NPs decreased significantly, while DOX-CCA NPs were less affected. The results of these quantitative analyses further verified that LA modification could enhance the eight capabilities of nanoparticles on cancer cells, thereby enhancing the drug delivery efficiency.

**FIGURE 5:**
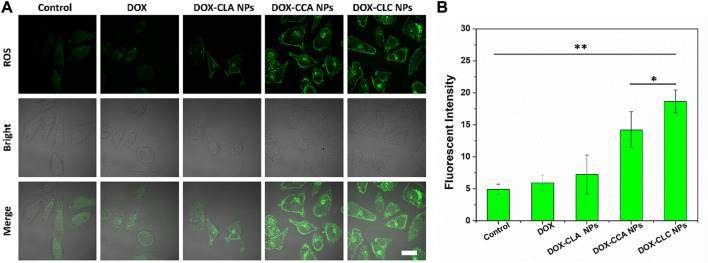
*In vitro* ROS evaluation of each sample in HepG2 cells observed by CLSM, scale bar = 10 um **(A)**; Quantitative analysis of the ROS fluorescence intensity measured by ImageJ **(B)**; * represents *p* < 0.05; ** represents *p* < 0.01.

Next, *in vitro* ROS generation of each sample in HepG2 cells was detected by the ROS kit. As shown in Figure 5A, there was only a very weak green fluorescence in the control cells, indicating that the ROS content in the cancer cells was low. However, the intracellular green fluorescence signals of DOX and DOX-CLA NPs treated cells were still weak, indicating that DOX and DOX-CLA NPs did not have significant ROS generation capacity. In contrast, DOX-CCA NPs and DOX-CLC NPs-treated cells produced a large amount of green fluorescence, indicating that CA could be shed from the nanoparticles and generate ROS inside the cells, thereby amplifying the oxidative stress in cancer cells. Figure 5B is a quantitative analysis of intracellular ROS content. Obviously, the intracellular ROS content of CA-contained nanoparticles (DOX-CCA NPs and DOX-CLC NPs) was much higher than that of control, free DOX and DOX-CLA NPs. DOX-CLC NPs generated the largest amount of ROS in cells, which was due to the LA-promoted cellular uptake of nanoparticles. These results were consistent with the results in Figure 4.

### 3.4 *In vitro* cytotoxicity

**FIGURE 6:**
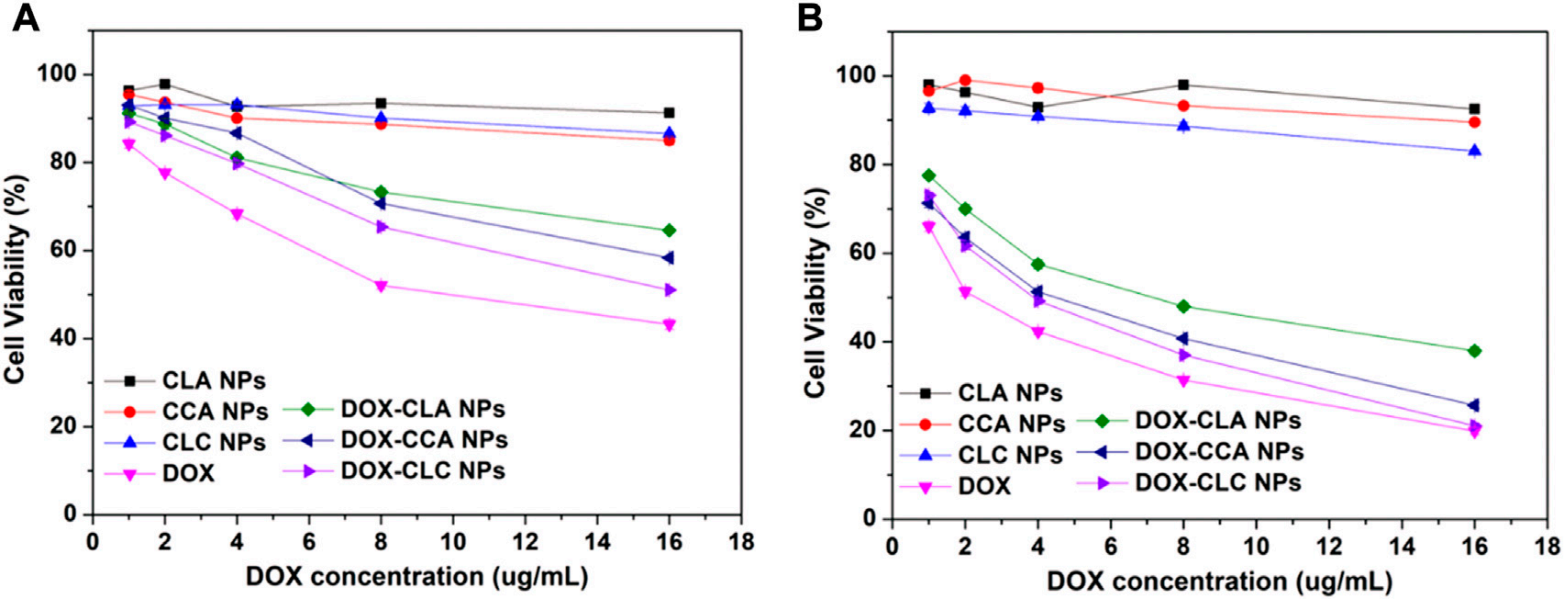
Cytotoxicity of each sample after co-cultured with H22 cells **(A)** and HepG2 cells **(B)** for 48 h.

Next, the cytotoxicity of free DOX, DOX-loaded nanoparticles and corresponding empty nanoparticles toward H22 and HepG2 were then measured by MTT assay. Cell viability after co-incubating with all these samples at various concentrations for 48 h was presented in Figure 6. These nanoparticles showed different degrees of cytotoxicity to H22 and HepG2 cells. For H22 cells, the cell viability after cocultured with CLA NPs, CCA NPs and CLC NPs for 48 h was 91.2%, 84.9%, and 86.7%, respectively. CCA NPs and CLC NPs show higher cytotoxicity than CLA NPs. This may be due to the cleavage of Schiff bases in the acidic environment in the cells and the release of cinnamaldehyde from the nanoparticles. CA can induce the production of ROS and inhibits the activity of cancer cells. After co-incubating with free DOX and DOX-loaded nanoparticles for 48 h, the cell viability decreased to 43.2% (free DOX), 64.6% (DOX-CLA NPs), 58.3% (DOX-CCA NPs), and 51.1% (DOX-CLC NPs). CA can induce apoptosis of cancer cells by generating ROS, it has a synergistic effect with DOX. Therefore, DOX-CCA NPs and DOX-CLC NPs are more cytotoxic than DOX-CLA NPs. The cell viability of CLA NPs, CCA NPs and CLC NPs treated HepG2 cells was 92.6%, 89.6%, and 83.0%, which is consistent with the results of H22 cells. However, the survival rate of HepG2 cells treated with free DOX and DOX-loaded nanoparticles is lower than that of the corresponding H22 cells, which indicates that HepG2 cells are more sensitive to the toxicity of DOX. At the end of the experiments, the cell viability of free DOX, DOX-CLA NP, DOX-CCA NPs, and DOX-CLC NPs treated HepG2 cells was 19.9%, 37.9%, 25.7, and 21.1%, respectively. In addition, the cytotoxicity of the three DOX-loaded nanoparticles against H22 cells and HepG2 cells are lower than that of free DOX, which may be caused by the controlled drug release rate of the nanoparticles in the cell.

### 3.5 Growth inhibition in tumor like multicellular spheroids

**FIGURE 7:**
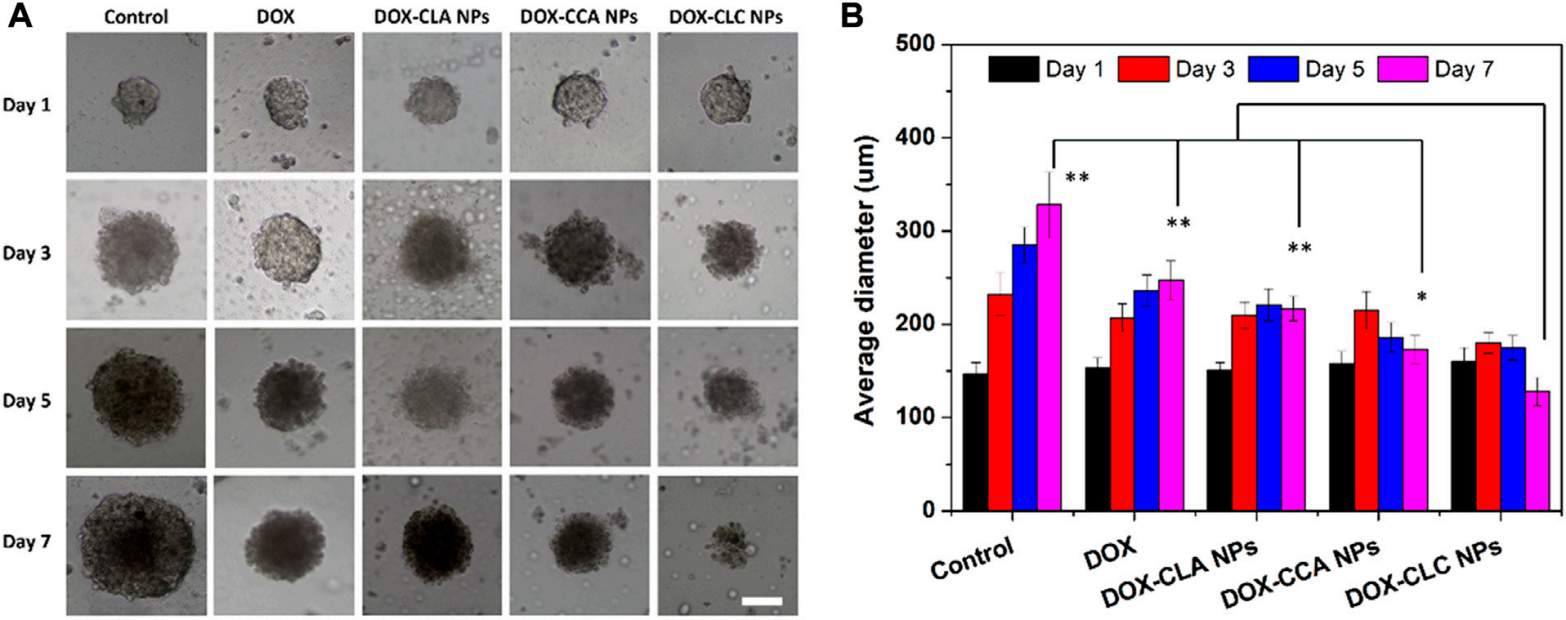
The growth inhibition of HepG2 MCs after co-incubating with free DOX, DOX-CLA NPs, DOX-CCA NPs and DOX-CLC NPs for 7 days **(A)**, scale bar = 100 um; The average diameter changes of MCs during the treatment were measured by ImageJ, *n* = 3 **(B)**; * represents *p* < 0.05, ** represents *p* < 0.01 at day 7.

In order to investigate the growth inhibitory efficiency of the prepared nanoparticles, HepG2 MCS were collected and co-cultured with free DOX, DOX-CLA NPs, DOX-CCA NPs, and DOX-CLC NPs for a week. HepG2 MCS cultured in a fresh culture medium without any drug were set as control. Figure 7A shows the representative images of HepG2 MCS co-cultured with each sample. For control group (MCS cultured with fresh medium), cells in outer layer of MCS grew rapidly during treatment. As a result, the diameter and volume of MCS continued to increase from day 1 to day 7. On the opposite, free DOX showed a certain inhibitory ability to the growth of MCS. The diameter and volume of the MCS slightly increase, and then remain unchanged. This indicates that DOX can effectively inhibit the proliferation of the periphery cells of MCS, thereby inhibiting the growth of MCS. DOX could accumulate at the few outer layer cells of MCs and inhibit the growth of MCs in a certain extent. However, cells in middle and center of MCs grew tightly with the secretion of ECM, hindered the dispersion of DOX and compromised the therapeutic effect. DOX-CLA NPs and DOX-CCA NPs showed similar inhibition results as DOX. MCS treated with DOX-CLC NPs showed the best anti-tumor activity. The outer cells of MCS continue to die due to the toxicity of DOX and CA, which eventually leads to the continuous reduction of the diameter and volume of MCS within 7 days. Figure 7B shows the average diameter changes of MCs during the treatment. The change trend of the average size of MCs in the Control group was consistent with that in Figure 7A, increasing from 147 ± 12 μm (Day 1) to 328 ± 35 μm (Day 7). DOX slowed down the growth of MCs to some extent, but the average diameter of MCs still reached 247 ± 21 μm on Day 7. Besides, the average diameter of DOX-CLA NPs and DOX-CCA NPs treated MCs reached to 217 ± 13 μm and 173 ± 15 μm at day 7, respectively. DOX-CLC NPs exhibited the most significant ability to inhibit the growth of MCs, and the average diameter of MCs decreased to 128 ± 15 μm on Day 7. All these results prove that DOX-CLC NPs can improve the uptake and enrichment of nanoparticles in cells through the targeting function of LA, and further release DOX and CA, producing a synergistic anti-tumor effect.

### 3.6 *In vivo* antitumor effect of DOX-CLC NPs

**FIGURE 8:**
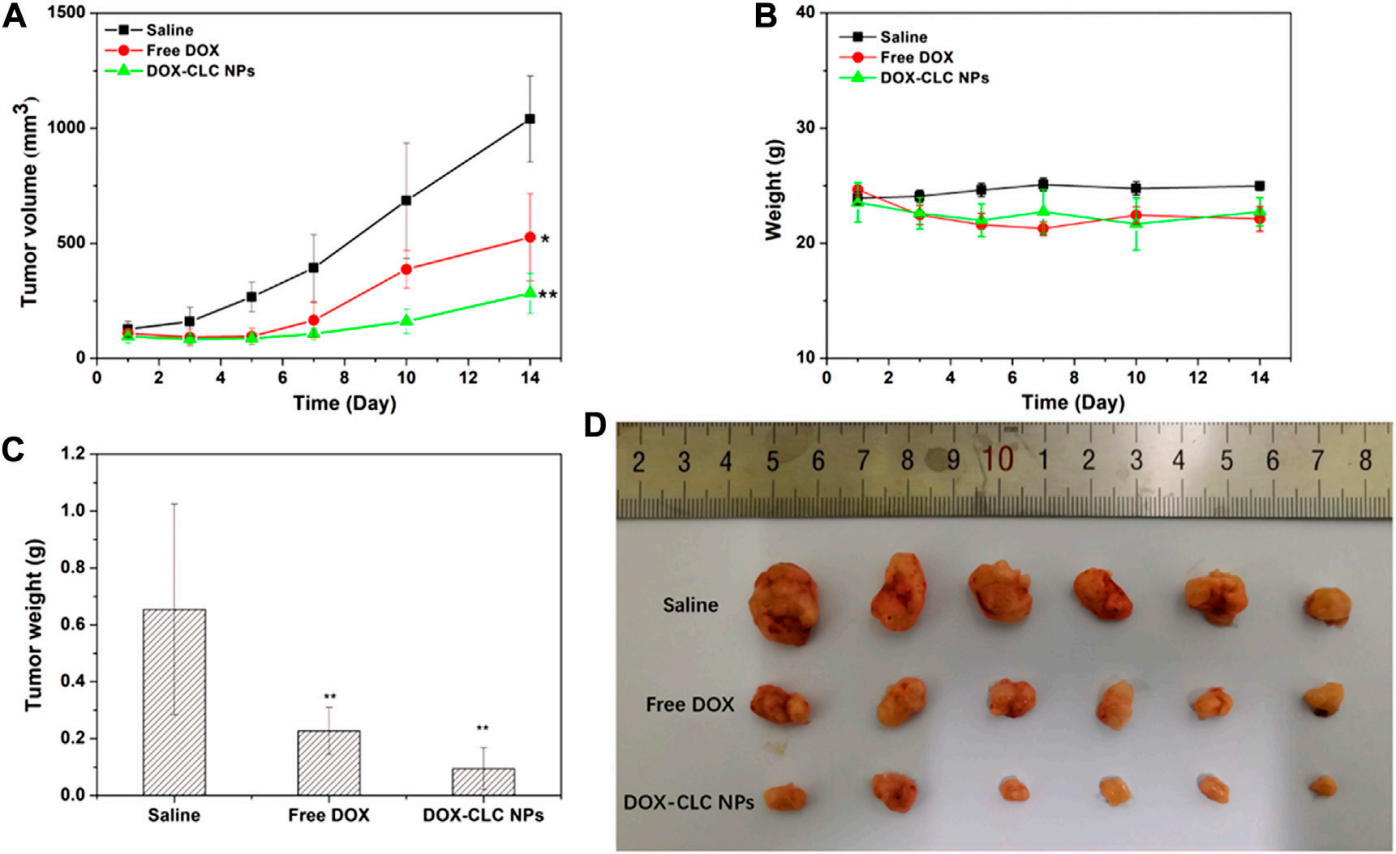
H22 tumor-bearing mice were treated with saline (control), free DOX and DOX-CLC NPs for 14 days. Tumor volume change during the treatment **(A)**; Mice bodyweight change during the treatment **(B)**; Tumor weight after treating with saline, free DOX and DOX-CLC NPs for 14 days **(C)**; The tumor image at the end of the treatment **(D)**. * represents *p* < 0.05 versus saline; ** represents *p* < 0.01 versus saline.

**FIGURE 9:**
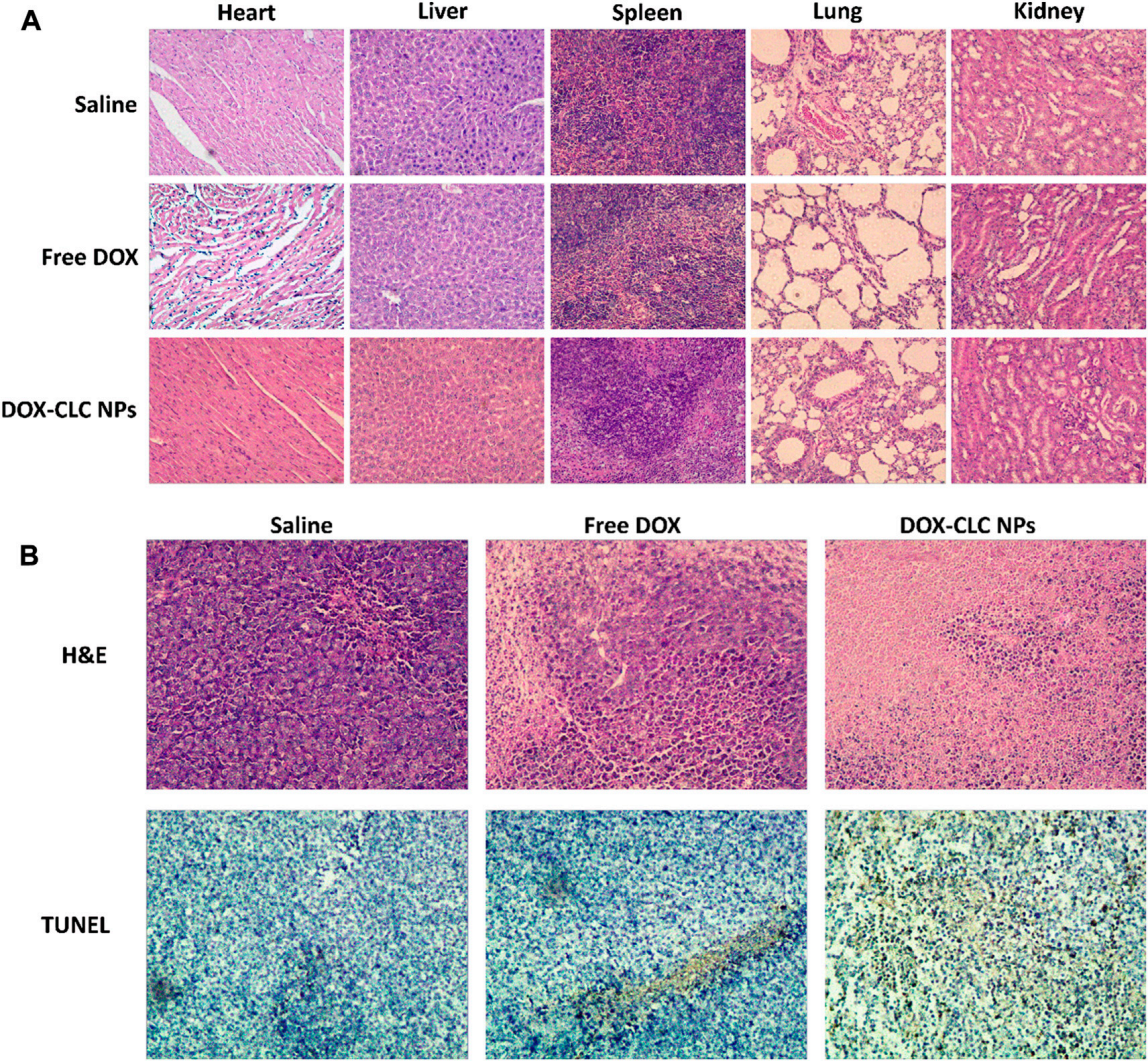
Histopathological analysis of heart, liver, spleen, lung, and kidney sections stained with H&E at day 14 **(A)**; H&E and TUNEL analysis of tumor at day 14 **(B)**; Images were obtained under an inverted microscope using a ×20 objective.

According to the results of cell experiments, it was found that among the three drug-loaded nanoparticles, DOX-CLC NPs had the best anti-tumor effect. Therefore, we used a mouse liver cancer model to preliminarily verify the anti-tumor activity of DOX-CLC NPs *in vivo*. Free DOX and DOX-CLC NPs were administrated in H22 tumor-bearing mice *via* tail vein while the tumor volume reached to 100 mm^3^. Figures 8A,B show the change of tumor volume and mice body weight during the 14 days of treatment. On the 14th day, the average tumor volume of saline treated mice increased to 1,040 ± 187 mm^3^, while the average tumor volume of free DOX-treated mice increased to 526 ± 189 mm^3^. For DOX-CLC NPs treated mice, the tumor volume has no significant change in the initial 7 days, indicating that DOX-CLC NPs almost inhibit the growth of H22 tumors. At the end of the experiment, the mean tumor volume of DOX-CLC NPs treated mice only grew to 283 ± 86 mm^3^. In addition, free DOX-treated mice show a slight decrease in body weight, which is may due to the toxicity of DOX. On 14th day, all these mice were sacrificed by cervical dislocation. The tumors of the mice were taken out and weighed. The results are shown in Figures 8C,D. It can be seen from Figure 8D that the tumors of the mice treated with saline are very large, and DOX effectively inhibited the growth of tumors. The mice treated with DOX-CLC NPs had the smallest tumors. The average weight of saline, free DOX and DOX-CLC NPs treated mice were 0.65 ± 0.37, 0.23 ± 0.08, and 0.094 ± 0.07 g, respectively. The tumor growth inhibition rate is 64.6% (DOX) and 85.5% (DOX-CLC NPs). Figure 9A shows the H&E stained sections of major organs, such as the heart, liver, spleen, lungs, and kidneys. The results demonstrate that DOX-CLC NPs do not show any significant damage in these organs, whereas free DOX showed significant cardiotoxicity. Tumor apoptosis was studied using histopathological analysis and TUNEL analysis. The tumor cell nucleus apoptosis of the DOX-CLC NPs treated group was more remarkable than that of the free DOX and control groups (Figure 9B). These results directly demonstrate that the synergistical effect between DOX and ROS. Furthermore, H&E stained sections of major organs, such as the heart, liver, spleen, lungs, and kidneys, from each group were investigated. The results demonstrate that DOX-CLC NPs do not show any significant damage in these organs. The *in vivo* antitumor study further demonstrated that DOX-CLC NPs possess an excellent synergistic antitumor effect.

## 4 Conclusion

Herein, a kind of multifunctional nanoparticles with the tumor-targeting ability and combination chemotherapy were prepared by LA modified CS and CA modified CS. *In vitro* MCS inhibition and *in vivo* antitumor study demonstrated that DOX-CLC NPs have better anti-tumor effects than free DOX. This may be caused by the following reasons: 1) Nanoparticles have good stability, which effectively improves the stability and half-life of DOX in blood circulation. 2) Nanoparticles are passively enriched in tumor tissues through the EPR effect, and LA modification further improves the ability of nanoparticles to be taken up by tumor cells. 3) In tumor cells, nanoparticles depolymerize and release DOX and CA; CA induces cells to produce ROS, amplifies the oxidative stress pressure of tumor cells, and promotes apoptosis; DOX directly produces cytotoxicity and has a good synergistic anti-tumor ability with CA.

## Data Availability

The original contributions presented in the study are included in the article/supplementary material, further inquiries can be directed to the corresponding authors.
